# Foot Health Status Questionnaire (FHSQ) in Spanish People with Type 2 Diabetes Mellitus: Preliminary Values Study

**DOI:** 10.3390/ijerph17103643

**Published:** 2020-05-22

**Authors:** Francisco Javier Domínguez-Muñoz, Miguel Angel Garcia-Gordillo, Rodrigo Anibal Diaz-Torres, Miguel Ángel Hernandez-Mocholi, Santos Villafaina, Daniel Collado-Mateo, Carmen Jiménez-Fernández, Dimas Igual-Fraile, Fernando Pérez-Escanilla, Giovanna Martín-Gómez, José Carmelo Adsuar, Narcis Gusi

**Affiliations:** 1 Physical Activity and Quality of Life Research Group (AFYCAV), Faculty of Sport Science, University of Extremadura, 10003 Cáceres, Spain; fjdominguez@unex.es (F.J.D.-M.); rodrigodiaztorres92@gmail.com (R.A.D.-T.); mhmocholi@unex.es (M.Á.H.-M.); svillafaina@unex.es (S.V.); ngusi@unex.es (N.G.); 2 Facultad de Administración y Negocios, Universidad Autónoma de Chile, Sede Talca 3467987, Chile; 3 Centre for Sport Studies, Rey Juan Carlos University, 28943 Fuenlabrada, Spain; danicolladom@gmail.com; 4 Primary Care Center “Manuel Encinas”, 10001 Cáceres, Spain; carmenjife@gmail.com (C.J.-F.); dimasigual@gmail.com (D.I.-F.); gimago_87@hotmail.com (G.M.-G.); 5 Primary Care Center SACYL, 37001 Salamanca, Spain; fpereze@semg.es; 6 Health Economy Motricity and Education (HEME) Research Group, Faculty of Sport Science, University of Extremadura, 10003 Cáceres, Spain; jadssal@unex.es; 7 CIBER de Fragilidad y Envejecimiento Saludable, 28029 Madrid, Spain

**Keywords:** type 2 diabetes, FHSQ, feet, health related quality of life

## Abstract

Background and objectives: Type 2 Diabetes Mellitus (T2DM) is a chronic disease characterized by hyperglycemia. T2DM affects millions of people, and has a lot of complications such as impaired sensation in the feet. Moreover, it is important to know the health of the feet of people with T2DM. The aim of this study is to know the preliminary values of the Foot Health Status Questionnaire (FHSQ) in people with T2DM. Materials and Methods: A total of 87 patients with T2DM with an average age of 65.56 years, divided in 54 men and 33 women, participated in this cross-sectional study. The main outcome was the health of the foot as measured by the FHSQ questionnaire. This questionnaire collects data on eight dimensions: Foot Pain, Foot Function, Shoe, General Foot Health, General Health, Physical Activity, Social Capacity, and Vigor. Results: Patients with T2DM have lower values in Foot Pain; median values in General Foot Health and high values in Foot Function, Shoe, Physical Activity and Social Capacity. Some of these dimensions are affected by age, diabetes control, Body Mass Index (BMI), and years of diagnosis. Females with T2DM have more problems than males in the Shoe, General Foot Health, Physical Activity and Vigor dimensions. Conclusions: this research gives us preliminary values of the FHSQ in Spanish patients with T2DM and divided by gender, age, diabetes control, BMI, and years of diagnosis in people with T2DM.

## 1. Introduction

Diabetes Mellitus (DM) is a chronic disease characterized by hyperglycemia [1]. This disease appears when the pancreas does not secrete enough insulin to control blood glucose. Insulin is a hormone secreted by the pancreas whose function is to regulate blood sugar levels [2]. DM is a global public health issue that currently affects approximately 415 million people in the world. It is estimated that this number will increase to 642 million people in 2040, with an interval between 521–829 million people [3]. People with DM present high glucose levels, with values above 200 mg/dL. Due to repeated hyperglycemia, it can lead to the disturbance of different organs and systems of the human body, blood vessels, and nerves [4].

DM is related to a series of complications, such as neuropathy. Peripheral neuropathy is defined as “peripheral, somatic, or autonomic nerve damages”, and it consists of various and different clinical entities that include diffuse neuropathies (distal symmetrical sensorimotor polyneuropathy) and focal neuropathies (entrapment, mononeuropathy, plexopathy, radiculopathy, and cranial neuropathy). In this regard, DM is the most frequent cause of peripheral neuropathy, which is mainly a sensory neuropathy. This is initially presented in the distal region of the inferior extremities [5], affecting 50% of the people that suffer from long-term DM [6].

The most important neuropathic alteration regarding diabetic foot is the loss of sensation since it becomes exposed to mechanical, chemical, or thermal painless trauma [7]. Frequently, the problems begin due to “domestic surgery,” i.e., when patients cut their nails and calluses too deep since they no longer have sensitivity in that region, predisposing them to acquire the infection easily [8]. On the other hand, the subject with DM can cause injuries to himself or feel the manifestation of these while trying to heat their feet with hot water as a response to the sensation of cold feet [9]. In addition, the incorrect use of footwear that forces defective plantar support produces traumatic injuries, especially if the neuropathy already produced neuro-arthropathies [10]; the resulting axis deviation of the bones deforms the foot, increases its diameter, and widens it [8]. By perpetuating the use of the same damaging footwear, which is tolerated due to insensitivity, a vicious cycle begins [11]. The situation is aggravated with edema, inflammation, and sometimes painful infection. The deformed position of the toes is common: claw or trigger toes, especially if the intrinsic muscles of the foot have been affected; furthermore, these deformations can produce new calluses [8].

The Foot Health Status Questionnaire (FHSQ) is a valid and trustworthy instrument about health quality regarding specific foot health, initially developed to assess the results of surgical treatment of common foot diseases [12]. However, it has been validated in different podiatric diseases as well, such as skin, neurological, and musculoskeletal diseases, and to determine the efficacy of foot orthoses [13,14]. The questionnaire is used in researches, where people with DM or people that have ulcers related to DM participated [15,16]. There is a study in the Spanish sample, where FHSQ scores between people with type 1 DM and T2DM were compared [17].

Some reasons are behind the use of the FHSQ questionnaire: (1) this instrument is easy to administer and complete [18], (2) this questionnaire is one of the most widely used and validated measures of foot pain and disability [12], (3) this questionnaire has been proposed as an outcome measure in clinical trials of foot disorders in patients with type 2 MD because of its responsiveness [19].

According to our knowledge, there is not any study where this questionnaire’s preliminary values by sex in Spanish patients with T2DM have been published. Therefore, the objective of this research was to report the descriptive values of the FHSQ in Spanish patients with T2DM.

## 2. Materials and Methods

### 2.1. Research Design

The research design is a cross-sectional study.

### 2.2. Participants

**Figure 1:**
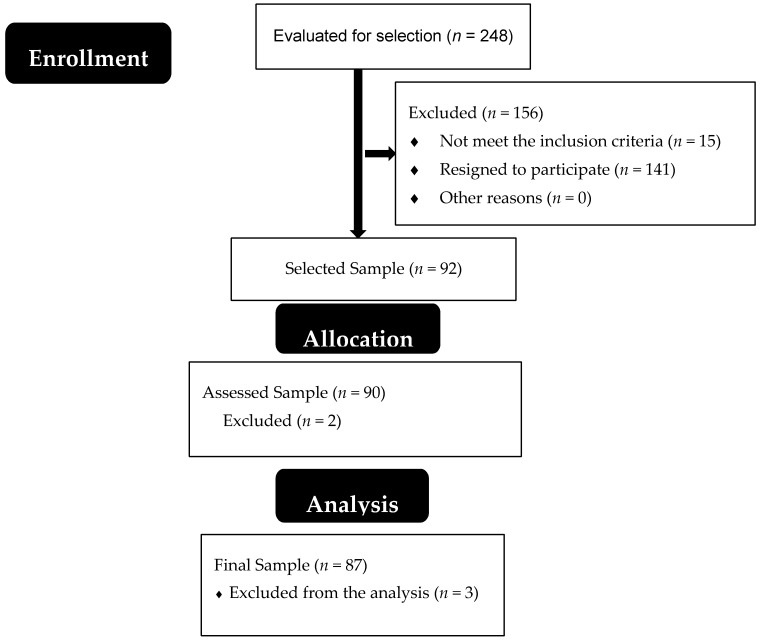
Flow diagram.

The sample was composed of 87 people with T2DM Figure 1. The sample was recruited from the Primary Care Center “Manuel Encinas” from the city of Caceres in Extremadura, Spain. The inclusion criteria to participate in the research were: (a) having a diagnosis of T2DM; (b) having read and signed the informed consent. The research’s exclusion criteria were: (a) being pregnant; (b) taking any psychotropic or chemotherapy medication; (c) suffering from other diseases related to balance and walking, such as Parkinson; (d) having a diagnosis of diabetic peripheral neuropathy in an advanced stage. The research protocol was approved by the Bioethical Committee of the Universidad de Extremadura (44/2012), taking into account the Helsinki declaration and the national legislation regarding bioethics, biomedical research, and sample confidentiality.

### 2.3. Measurement Tools

The FHSQ is composed of 3 sections. Each section includes a number of questions. The first section includes 13 questions and evaluates four dimensions (Foot Pain, Foot Function, Shoe, and General Foot Health). The second section includes 20 questions and evaluates the four dimensions left (General Health, Physical Activity, Social Capacity, and Vigor). Each question is valued following a Likert-type scale, and the scoring goes from 0 to 100, where 0 is the worst foot health status and, 100 is the best possible foot health status. Lastly, the third section refers to the socio-demographic values of the sample. The questionnaire has been translated and adapted to Spanish, showing adequate psychometric properties regarding construct and trustworthiness validity.

A stadiometer (Seca 22, Hamburg, Germany) was used to measure the height and weight. Body Mass Index (BMI) was calculated with the following formula: weight (kg)/height^2^ (m).

With regard to obtaining height and weight, we have followed the following recommendations [20]:

- Do not wear shoes.
- The subject must have his feet and heels together.
- He should touch the measuring rod with his buttocks and back.
- The head should be in a neutral state, looking forward.

A bioimpedanciometer Tanita Body Composition Analyzer (TANITA BC-418MA) was used to evaluate the body fat percentage.

As for the bioimpedance measurement procedure, we follow the following indications [21]:

- No intense physical exercise 24 h before.
- Urinate before the measurements.
- Measure weight and height at each evaluation.
- Settle in the supine position for 8-10 min beforehand.
- Correct position of the electrodes.
- Arms and legs should be separated from the trunk.
- Remove metallic elements.
- Report situations such as marked abdominal obesity, muscle mass, weight loss, menstrual cycle, and menopause.

### 2.4. Statistical Analysis

Means, medians, standard deviation, and interquartile ranges were calculated for the entire sample. These were segregated by sex and taking DM control, normal weight, and age into account. In regard to DM control, the sample was divided into two groups: one group with inadequate DM control and Glycated Hemoglobin (Hb1Ac) higher than or equal to 7%, and a group with Hb1Ac lower than 7% [22]. In order to calculate the groups with normal weight and overweight-obesity, the BMI values were used; these values were lower than 25 kg/m^2^ within the normal weight group and higher than or equal to 25 kg/m^2^ within the overweight-obesity group [23]. Age groups were divided between the ones that are younger than 65-years-old and the ones that are equal to or older than 65-years-old [24]. Lastly, we included two divisions for the years of diagnosis of the disease. In one division there was a group that had been diagnosed 5 years ago or less and another one above 5 years since the diagnosis of T2DM. In the other division there was a group that had been diagnosed less than 10 years ago and another group that had been diagnosed 10 years or more since the diagnosis of T2DM.

The Kolmogorov–Smirnov test was used to do the data distribution. The following variables followed a parametric distribution: age, weight, height, body fat percentage, and BMI; the remaining variables did not follow a parametric distribution. A Student’s t-test was used to check the statistically significant differences according to sex regarding the parametric variables. A Mann–Whitney U test was used for the non-parametric variables.

Regarding the different comparisons conducted between the dimensions of the FHSQ, a Mann–Whitney U test was used. In order to interpret the values obtained through the FHSQ, the following self-developed classification was used: from 0 to 24.9 is very low; from 25 to 49.9 is low; from 50 to 74.4 is medium; and from 75 to 100 is high. These analyses have been conducted through the statistical package SPSS 21.0 (SPSS Inc., Chicago, IL, USA).

## 3. Results

### 3.1. Participant Characteristics

Table 1 shows the characteristics of a sample of 87 people with an average age of 65.56 years and an average weight of 80.60 kg, presenting statistically significant differences in weight, height, and body fat percentage. Regarding BMI, the total is equal to 29.55 kg/m^2^, and the body fat percentage is equal to 33.11%; in this case, the women’s body fat percentage (39.58%) is higher than men’s (29.16%).

The number of years since the diagnosis in the total sample is equal to 9.71 years, and the number of painful years is equal to 1.86 years. Concerning the pain scale, the total sample’s score is equal to 3.89. This number is higher among women (6.12) than men (2.53).

### 3.2. Preliminary FHSQ Values

Table 2 shows the descriptive values of the FHSQ’s total sample. In Table 3 and Table 4, these data are segregated according to sex. Each table is divided in accordance with diabetes control, normal weight, age, and years of diagnosis. In the general table, high values can be observed regarding Foot function (94.39), Shoe (75.76), Physical Activity (80.49), and Social Capacity (91.23). The General Foot Health (57.67), General Health (66.66), and Vigor (67.45) dimensions show medium values.

Women have worse statistically significant scores regarding the Shoe, General Foot Health, Physical Activity, and Vigor variables. As a matter of fact, their scores for the Shoe and Physical Activity dimensions are placed at the medium level, while men’s scores regarding those dimensions are placed at the high level. Additionally, women’s scores regarding the General Foot Health dimension are placed at the low level, while men’s scores are placed at the medium level. The scores regarding the Vigor dimensions remain at the medium level.

Regarding Diabetes control in the total sample, people that have worse DM control show statistically significant differences within the General Foot Health dimension, while people with better diabetes control show better scores within this dimension. This data shows the same tendency among men, unlike the women’s case.

Regarding the normal weight criteria in the total sample, overweight people show statistically significant differences in the scores from the Foot Pain, Foot Function, and General Foot Health dimensions. In the men group, this only remains like this within the Foot Pain dimension, while, among women, there are no differences in any dimensions.

In the age category, there are no statistically significant differences in any dimensions of the total sample. However, there are statistically significant differences within the men group regarding the Physical Activity dimension, and within the women group regarding the Shoe and General Health dimensions.

Lastly, in the category of years of T2DM diagnosis, in the division of 5 or more years since T2DM diagnosis there are only statistically significant differences in Foot Function and General Foot Health in the subgroup of men. Meanwhile in the division of 10 years since the diagnosis of T2DM there are statistically significant differences in the General Foot Health in both the general sample and men. In contrast, in women, there are no statistically significant differences in any of the dimensions.

## 4. Discussion

According to our knowledge, this is the first research that offers FHSQ’s preliminary values with data segregated by sex regarding people with T2DM. Although the FHSQ has been previously applied in people with DM and—to date—there is only one research that has values for each dimension of the questionnaire regarding this kind of population, there are no preliminary values with data segregated by sex for the FHSQ regarding people with T2DM.

In Spain, the FHSQ has been applied to pregnant women [25], menopausal and non-menopausal women [26], college students [27], 6 to 12-year-old children [28], and seniors [29].

Concerning the dimensions of the questionnaire, the general data allow us to observe that in the case of menopausal and non-menopausal women, they show higher pain regarding the Foot Pain dimension than the subjects with T2DM, and vice-versa regarding the Foot Function, Shoe, General Foot Health, Social Capacity, and Vigor dimensions.

These values are consistent with the ones found in the article about pregnant women [25]. In comparison with college students [27], the same as before happens, i.e., Foot Pain is higher among people with T2DM. Regarding the research with 6 to 12-year-old children [28] with a normal arch foot, we can observe that patients with T2DM have worse scores regarding the Foot Pain, General Foot Health, General Health, Physical Activity, and Vigor dimensions. Lastly, when comparing our results with the article about seniors [29], the values were the subjects with T2DM have the worse score is in the Foot Pain dimension, while the best score is in the Shoe dimension; this is consistent with the previous articles.

About the sample size, our research has 87 people with T2DM, while in other articles, the sample is smaller, being 61 participants in the studies by Burns et al. [16], and 62 participants in the research of Palomo-López et al. [17]. Concerning the data of the dimensions of the FHSQ, in the study by Burns et al. [16], the values of the Foot Pain dimension among participants with experimental insoles were higher (47.10) than in our research. By contrast, the values among the sample with fake insoles (38.8) have a similar value to ours (37.99). Regarding the Foot Function dimension, the values from our research (95.13) are considerably higher than those present in this article: 45.20 within the group with experimental insoles and 47.40 within the group with fake insoles. This difference could be due to these patients having foot pathologies. About the data of the research by Palomo-López et al. [17], the values are considerably higher than ours regarding the Foot Pain dimension (78.75). By contrast, the dimensions that have a lower value in comparison to our research are Foot Function (78.12), Shoe (50.00), General Health (50.00), and Vigor (56.25). The remaining dimensions showcase similar values.

Regarding the statistically significant differences, these can be observed between men and women within the Shoe, General Foot Health, Physical Activity, and Vigor dimensions. There are also differences within the conditions “Diabetes Control” and “Overweight according to BMI” regarding the General Foot Health dimension. It should be noted that the General Foot Health dimension exhibits differences both in men and women. Within women, alongside the Shoe dimension, there are differences regarding the condition “Age”; however, these dimensions improve with age. We believe that this could be since women that are younger than 65 years old still use footwear with high heels with aesthetic goals [30], while women that are older than 65 years old prioritize commodity before aesthetic and decrease their use with age [31]. Regarding the General Health dimension, women over 65 have statistically significantly better overall health. This result may be due to the effect that retirement could have on having more time to care for oneself. These data are important for future research since they can allow proposing studies taking the preliminary values presented in this research into account and designing physical activity programs oriented to this population so that these dimensions do not worsen further because of the sickness.

The present research has a series of limitations. One of them is the number of the total sample since it was determined through convenience sampling, which is not representative of the Spanish population. On the other hand, the sample focused on Spanish residents and did not take into account the possible influence generated by cultural differences of foreign participant subjects. Lastly, when the sample has been divided according to different criteria (age, sex, BMI, and years of diagnosis), the number of participants is reduced. This leads to a reduction in statistical power.

## 5. Conclusions

In conclusion, this research gives us preliminary values of the FHSQ in Spanish patients with T2DM. In this sample, high values can be observed regarding Foot function, Shoe, Physical Activity, and Social Capacity. The General Foot Health, General Health, and Vigor dimensions show medium values. These data could be useful to compare the foot health status of people with T2DM that go to primary care centers until an epidemiological study with representative values is conducted.

## Figures and Tables

**Table 1:** Participant characteristics.

	Total (*n* = 87)		Men (*n* = 54)		Women (*n* = 33)		*p*
	Mean	SD	Mean	SD	Mean	SD	
**Age (years)**	65.56	8.74	65.55	8.31	65.57	9.52	0.827 *
**HB1Ac (%)**	6.74	1.01	6.79	0.95	6.66	1.10	0.430 ^†^
**Weight (kg)**	80.60	16.23	85.64	17.21	72.35	10.19	< 0.001 *
**Height (cm)**	164.83	10.12	170.06	7.81	156.27	7.25	< 0.001 *
**BMI (kg/m^2^)**	29.55	4.43	29.50	4.81	29.64	3.80	0.523 *
**Body Fat Percentage**	33.11	7.31	29.16	5.04	39.58	5.67	< 0.001 *
**Years of diagnosis**	9.71	8.80	9.11	7.26	10.70	10.93	0.809 ^†^
**Years of pain**	1.86	0.40	1.85	0.45	1.87	0.33	0.937 ^†^
**Pain (0–10)**	3.89	14.98	2.53	9.98	6.12	20.74	0.631 ^†^

SD: Standard Deviation; Hb1Ac: Glycated Hemoglobin; BMI: Body Mass Index; * *p* of t of Student; ^†^ *p* of the U of Mann–Whitney.

**Table 2:** Preliminary values of the Foot Health Status Questionnaire (FHSQ) in patients with Diabetes Mellitus Type 2 (*n* = 87).

	Foot Pain	Foot Function	Shoe	General Foot Health	General Health	Physical Activity	Social Capacity	Vigour
**General**								
Median (IQR)	29.20 (7.80)	100.00 (0.00)	100.00 (33.33)	60.00 (30.00)	70.00 (40.00)	88.88 (22.22)	100.00 (0.00)	68.75 (37.50)
Mean (SD)	38.59 (22.89)	94.39 (16.31)	75.76 (38.43) ζ	57.67 (25.03) ζ	66.66 (23.16)	80.45 (22.13) ζ	91.23 (20.71)	67.45 (23.86) ζ
Range	21.50–100.00	12.50–100.00	0.00–100.00	0.00–100.00	10.00–100.00	5.55–100.00	12.50–100.00	0.00–100.00
**Diabetes Control**								
Hb1Ac < 7 (*n* = 61)								
Median (IQR)	29.30 (6.95)	100.00 (0.00)	100.00 (58.35)	60.00 (25.00)	70.00 (40.00)	88.88 (25.00)	100.00 (0.00)	68.75 (37.50)
Mean (SD)	37.97 (22.37)	94.26 (16.26)	72.81 (39.43)	60.65 (25.43) *	66.39 (24.15)	82.14 (18.26)	91.39 (19.70)	67.11 (24.60)
Range	21.50–100.00	12.50–100.00	0.00–100.00	0.00–100.00	10.00–100.00	27.77–100.00	12.50–100.00	0.00–100.00
Hb1Ac > 7 (*n* = 26)								
Median (IQR)	28.90 (11.25)	100.00 (0.00)	100.00 (12.49)	60.00 (31.25)	70.00 (32.50)	88.88 (22.22)	100.00 (0.00)	68.75 (39.06)
Mean (SD)	40.03 (24.46)	94.71 (16.74)	82.69 (35.74)	50.67 (23.03)	67.30 (21.08)	76.49 (29.36)	90.86 (23.33)	68.26 (22.49)
Range	24.40–100.00	25.00-100.00	0.00–100.00	0.00–100.00	30.00–100.00	5.55–100.00	25.00–100.00	12.5–100.00
**BMI Normal Weight (kg/m^2^)**								
BMI < 25 (*n* = 10)								
Median (IQR)	24.10 (15.81)	100 (0.00)	100 (29.16)	82.50 (25.00)	75.00 (30.00)	91.66 (19.44)	100.00 (0.00)	81.25 (40.62)
Mean (SD)	37.39 (29.12) *	99.37 (1.97) *	87.49 (17.67)	76.00 (14.68) *	71.00 (22.82)	88.88 (12.28)	100.00 (.00)	76.25 (19.93)
Range	21.50–100.00	93.75–100.00	58.33–100.00	60.00–100.00	30.00–100.00	66.66–100.00	100.00–100.00	50.00–100.00
BMI > 25 (*n* = 77)								
Median (IQR)	29.80 (7.75)	100.00 (0.00)	100.00 (54.16)	60.00 (17.50)	70.00 (40.00)	88.88 (22.22)	100.00 (0.00)	68.75 (37.50)
Mean (SD)	38.74 (22.19)	93.75 (17.23)	74.24 (40.17)	55.29 (25.17)	66.10 (23.29)	79.36 (22.93)	90.09 (21.77)	66.31 (24.20)
Range	25.00–100.00	12.50–100.00	0.00–100.00	0.00–100.00	10.00–100.00	5.55–100.00	12.50–100.00	0.00–100.00
**Age**								
< 65 years (*n* = 32)								
Median (IQR)	29.25 (8.80)	100.00 (0.00)	95.83 (66.66)	60.00 (51.87)	70.00 (30.00)	94.44 (22.22)	100.00 (0.00)	75.00 (34.37)
Mean (SD)	35.17 (15.84)	93.55 (19.60)	69.79 (39.18)	58.90 (29.35)	65.31 (23.68)	84.37 (21.28)	92.18 (19.50)	70.89 (20.85)
Range	21.50–84.37	12.50–100.00	0.00–100.00	0.00–100.00	10.00–100.00	5.55-100.00	25.00–100.00	12.50–100.00
> 65 years (*n* = 55)								
Median (IQR)	29.20 (7.90)	100.00 (0.00)	100.00 (16.66)	60.00 (0.00)	70.00 (40.00)	83.33 (22.22)	100.00 (0.00)	68.75 (37.50)
Mean (SD)	40.58 (26.07)	94.88 (14.23)	79.24 (37.91)	56.95 (22.40)	67.45 (23.03)	78.18 (22.49)	90.68 (21.54)	65.45 (25.42)
Range	23.50–100.00	25.00–100.00	0.00–100.00	0.00–100.00	10.00–100.00	5.55–100.00	12.50–100.00	0.00–100.00
**Years of diagnosis**								
≤ 5 years (*n* = 36)								
Median (IQR)	29.55 (10.40)	100.00 (0.00)	100.00 (37.51)	60.00 (38.13)	70.00 (47.50)	88.88 (22.22)	100.00 (0.00)	78.12 (37.50)
Mean (SD)	39.57 (24.18)	98.61 (4.51)	78.01 (36.63)	64.65 (21.85)	66.39 (23.80)	83.33 (16.16)	92.36 (20.11)	69.79 (23.46)
Range	21.50–100.00	75.00–100.00	0.00–100.00	25.00–100.00	20.00–100.00	50.00–100.00	12.50–100.00	12.50–100.00
> 5 years (*n* = 51)								
Median (IQR)	29.20 (7.50)	100.00 (6.25)	100.00 (33.34)	60.00 (17.50)	70.00 (40.00)	88.88 (22.22)	100.00 (0.00)	68.75 (37.50)
Mean (SD)	37.90 (22.16)	91.42 (20.54)	74.18 (39.94)	52.74 (26.15)	66.86 (22.93)	78.43 (25.50)	90.44 (21.30)	65.80 (24.25)
Range	23.50–100.00	12.50–100.00	0.00–100.00	0.00–100.00	10.00–100.00	5.56–100.00	25.00–100.00	0.00–100.00
< 10 years (*n* = 52)								
Median (IQR)	28.65 (7.02)	100.00 (0.00)	100.00 (16.67)	60.00 (25.00)	70.00 (40.00)	88.88 (26.39)	100.00 (0.00)	68.75 (37.50)
Mean (SD)	37.78 (22.46)	96.03 (12.80)	81.24 (34.41)	64.27 (24.01) *	67.69 (23.23)	82.90 (19.14)	91.82 (20.54)	68.26 (22.91)
Range	21.50–100.00	31.25–100.00	0.00–100.00	0.00–100.00	20.00–100.00	5.55–100.00	12.50–100.00	12.50–100.00
≥ 10 years (*n* = 35)								
Median (IQR)	29.90 (8.30)	100.00 (6.25)	100.00 (91.67)	60.00 (35.00)	70.00 (30.00)	83.33 (22.22)	100.00 (0.00)	68.75 (43.75)
Mean (SD)	39.79 (23.80)	91.96 (20.42)	67.61 (42.95)	47.85 (23.54)	65.14 (23.31)	76.82 (25.82)	90.35 (21.24)	66.25 (25.50)
Range	24.40–100.00	12.50–100.00	0.00–100.00	0.00–85.00	10.00–100.00	5.56–100.00	25.00–100.00	0.00–100.00

IQR: Inter-Quartile Range; SD: Standard Deviation; Hb1Ac: Glycated Hemoglobin; BMI: Body Mass Index; N/A: Not Applicable. * Statistically significant differences in the parameter used for comparison with the U of Mann–Whitney; ζ Statistically significant differences between men and women performing with the U of Mann–Whitney.

**Table 3:** Preliminary values of the Foot Health Status Questionnaire (FHSQ) in men with Diabetes Mellitus Type 2 (*n* = 54).

	Foot Pain	Foot Function	Shoe	General Foot Health	General Health	Physical Activity	Social Capacity	Vigour
**General**								
Median (IQR)	28.95 (7.42)	100.00 (0.00)	100.00 (10.41)	60.00 (25.00)	70.00 (40.00)	91.66 (22.22)	100.00 (0.00)	75.00 (32.81)
Mean (SD)	37.99 (22.55)	95.13 (15.09)	85.18 (29.92)	63.37 (22.68)	69.07 (22.75)	83.95 (22.37)	92.36 (18.87)	72.56 (22.33)
Range	21.50–100.00	25.00–100.00	0.00–100.00	0.00–100.00	10.00–100.00	5.55–100.00	25.00–100.00	0.00–100.00
**Diabetes Control**								
Hb1Ac < 7 (*n* = 36)								
Median (IQR)	28.65 (5.47)	100.00 (0.00)	100.00 (22.91)	60.00 (25.00)	75.00 (30.00)	94.44 (16.66)	100.00 (0.00)	78.12 (35.93)
Mean (SD)	36.55 (21.58)	96.35 (12.17)	84.25 (29.12)	69.23 (19.68) *	71.11 (23.75)	88.27 (15.24)	94.05 (17.02)	72.56 (23.82)
Range	21.50–100.00	31.25–100.00	8.33–100.00	0.00–100.00	10.00–100.00	38.88–100.00	25.00–100.00	0.00–100.00
Hb1Ac > 7 (*n* = 18)								
Median (IQR)	29.20 (12.85)	100.00 (0.00)	100.00 (2.08)	60.00 (8.75)	70.00 (30.00)	86.11 (22.22)	100.00 (0.00)	71.87 (28.12)
Mean (SD)	40.85 (24.79)	92.70 (19.90)	87.03 (32.23)	51.66 (24.25)	65.00 (20.65)	75.30 (31.03)	90.97 (22.60)	72.56 (19.66)
Range	24.40–100.00	25.00–100.00	0.00–100.00	0.00–100.00	30.00–100.00	5.55–100.00	25.00–100.00	37.50–100.00
**BMI Normal Weight (kg/m^2^)**								
BMI < 25 (*n* = 7)								
Median (IQR)	23.80 (1.00)	100.00 (0.00)	100.00 (25.00)	85.00 (25.00)	80.00 (30.00)	100.00 (11.11)	100.00 (0.00)	87.50 (37.50)
Mean (SD)	34.48 (28.90) *	100.00 (0.00)	90.47 (16.96)	76.42 (16.25)	81.42 (14.63)	95.23 (6.75)	100.00 (0.00)	83.03 (17.57)
Range	21.50–100.00	100.00–100.00	58.33–100.00	60.00–100.00	60.00–100.00	83.33–100.00	100.00–100.00	56.25–100.00
BMI > 25 (*n* = 47)								
Median (IQR)	29.20 (8.00)	100.00 (0.00)	100.00 (8.33)	60.00 (12.50)	70.00 (40.00)	88.88 (16.66)	100.00 (0.00)	68.75 (31.25)
Mean (SD)	38.51 (21.79)	94.41 (16.07)	84.39 (31.45)	61.43 (22.98)	67.23 (23.28)	82.26 (23.41)	91.22 (20.00)	71.01 (22.70)
Range	25.60–100.00	25.00–100.00	0.00–100.00	0.00–100.00	10.00–100.00	5.55–100.00	25.00–100.00	0.00–100.00
**Age**								
< 65 years (*n* = 21)								
Median (IQR)	29.80 (11.30)	100.00 (0.00)	100.00 (25.00)	60.00 (25.00)	80.00 (35.00)	94.44 (11.11)	100.00 (0.00)	81.25 (25.00)
Mean (SD)	35.29 (15.64)	95.83 (15.09)	86.90 (24.23)	66.07 (26.21)	72.85 (21.94)	89.68 (20.43) *	95.23 (16.99)	76.48 (16.87)
Range	21.50–78.75	31.25–100.00	25.00–100.00	0.00–100.00	30.00–100.00	5.55–100.00	25.00–100.00	37.50–100.00
> 65 years (*n* = 33)								
Median (IQR)	28.60 (6.15)	100.00 (0.00)	100.00 (8.33)	60.00 (0.00)	70.00 (40.00)	88.88 (22.22)	100.00 (6.25)	68.75 (40.62)
Mean (SD)	39.70 (26.11)	94.69 (15.31)	84.09 (33.35)	61.66 (20.36)	66.66 (23.27)	80.30 (23.07)	90.53 (20.01)	70.07 (25.13)
Range	23.50–100.00	25.00–100.00	0.00–100.00	0.00–100.00	10.00–100.00	5.55–100.00	25.00–100.00	0.00–100.00
**Years of diagnosis**								
≤ 5 years *(n =* 20)								
Median (IQR)	28.10 (5.88)	100.00 (0.00)	100.00 (12.51)	78.75 (25.00)	80.00 (27.50)	94.44 (11.12)	100.00 (0.00)	81.25 (29.69)
Mean (SD)	38.31 (24.33)	100.00 (0.00) *	88.33 (24.24)	72.37 (19.54) *	73.00 (22.96)	89.72 (14.89)	97.50 (8.70)	76.56 (22.11)
Range	21.50–100.00	100.00–100.00	25.00–100.00	25.00–100.00	20.00–100.00	50.00–100.00	62.50–100.00	18.75–100.00
> 5 years (*n* = 34)								
Median (IQR)	29.20 (8,7)	100.00 (6.25)	100.00 (12.50)	60.00 (0.00)	70.00 (40.00)	88.88 (19.45)	100.00 (3.13)	68.75 (32.81)
Mean (SD)	37.80 (21.83)	92.28 (18.53)	83.33 (33.02)	58.09 (22.99)	66.76 (22.66)	80.55 (25.37)	89.34 (22.43)	70.22 (22.46)
Range	23.50–100.00	25.00–100.00	0.00–100.00	0.00–100.00	10.00–100.00	5.56–100.00	25.00–100.00	0.00–100.00
< 10 years *(n =* 32)								
Median (IQR)	28.60 (5.62)	100.00 (0.00)	100.00 (0.00)	60.00 (25.00)	75.00 (30.00)	94.44 (16.67)	100.00 (0.00)	81.25 (35.93)
Mean (SD)	37.18 (22.76)	96.87 (12.60)	87.50 (27.19)	69.53 (21.99) *	72.50 (21.99)	86.46 (19.75)	94.53 (16.78)	74.41 (20.78)
Range	21.50–100.00	31.25–100.00	8.33–100.00	0.00–100.00	20.00–100.00	5.56–100.00	25.00–100.00	18.75–100.00
≥ 10 years *(n =* 22)								
Median (IQR)	29.55 (10.87)	100.00 (6.25)	100.00 (27.08)	60.00 (0.00)	65.00 (32.50)	88.89 (19.44)	100.00 (15.62)	68.75 (29.69)
Mean (SD)	39.17 (22.74)	92.61 (18.16)	81.82 (33.89)	54.43 (21.04)	64.09 (23.43)	80.30 (25.76)	89.20 (21.58)	69.89 (24.67)
Range	24.40–100.00	25.00–100.00	0.00–100.00	0.00–85.00	10.00–100.00	5.56–100.00	25.00–100.00	0.00–100.00

IQR: Inter-Quartile Range; SD: Standard Deviation; Hb1Ac: Glycated Hemoglobin; BMI: Body Mass Index; N/A: Not Applicable. * Statistically significant differences in the parameter used for comparison with the U of Mann–Whitney.

**Table 4:** Preliminary values of the Foot Health Status Questionnaire (FHSQ) in women with Diabetes Mellitus Type 2 (*n* = 33).

	Foot Pain	Foot Function	Shoe	General Foot Health	General Health	Physical Activity	Social Capacity	Vigour
**General**								
Median (IQR)	29.90 (8.00)	100.00 (6.25)	83.33 (100.00)	60.00 (35.00)	70.00 (40.00)	77.77 (27.77)	100.00 (6.25)	56.25 (31.25)
Mean (SD)	39.57 (23.76)	93.18 (18.31)	60.35 (45.74)	48.33 (26.21)	62.72 (23.62)	74.74 (20.83)	89.39 (23.61)	59.09 (24.26)
Range	24.00–100.00	12.50–100.00	0.00–100.00	0.00–92.50	10.00–100.00	22.22–100.00	12.50–100.00	12.50–100.00
**Diabetes Control**								
Hb1Ac < 7 (*n* = 25)								
Median (IQR)	30.00 (7.80)	100.00 (6.25)	83.33 (100.00)	60.00 (35.00)	70.00 (35.00)	77.77 (19.44)	100.00 (18.75)	56.25 (28.12)
Mean (SD)	40.02 (23.77)	91.25 (20.72)	56.33 (46.60)	48.30 (27.97)	59.60 (23.53)	73.33 (18.90)	89.00 (23.19)	59.25 (24.01)
Range	24.00–100.00	12.50–100.00	0.00–100.00	0.00–92.50	10.00–100.00	27.77–100.00	12.50–100.00	12.50–100.00
Hb1Ac > 7 (*n* = 8)								
Median (IQR)	28.45 (9.07)	100.00 (0.00)	100.00 (75.00)	51.25 (33.75)	70.00 (45.00)	88.88 (36.11)	100.00 (0.00)	56.25 (40.62)
Mean (SD)	38.17 (25.29)	99.21 (2.20)	72.91 (43.35)	48.43 (21.42)	72.50 (22.51)	79.16 (27.01)	90.62 (26.51)	58.59 (26.71)
Range	25.00–100.00	93.75–100.00	0.00–100.00	25.00–85.00	40.00–100.00	22.22–100.00	25.00–100.00	12.50–93.75
**BMI Normal Weight (kg/m^2^)**								
BMI > 25 (*n* = 30)								
Median (IQR)	29.95 (7.55)	100.00 (6.25)	87.50 (100.00)	51.25 (35.00)	70.00 (35.00)	77.77 (34.72)	100.00 (15.62)	56.25 (32.81)
Mean (SD)	39.11 (23.17)	92.70 (19.14)	58.33 (47.24)	45.66 (25.79)	64.33 (23.58)	74.81 (21.77)	88.33 (24.55)	58.95 (25.03)
Range	25.00–100.00	12.50–100.00	0.00–100.00	0.00–92.50	10.00–100.00	22.22–100.00	12.50–100.00	12.50–100.00
**Age**								
< 65 years (*n* = 11)								
Median (IQR)	28.80 (6.40)	100.00 (6.25)	8.33 (75.00)	60.00 (35.00)	60.00 (40.00)	77.77 (44.44)	100.00 (25.00)	50.00 (31.25)
Mean (SD)	34.93 (16.98)	89.20 (26.52)	37.12 (42.38) *	45.22 (31.35)	50.90 (20.71) *	74.24 (19.91)	86.36 (23.35)	60.22 (24.25)
Range	24.20–84.37	12.50–100.00	0.00–100.00	0.00–92.50	10.00–70.00	44.44–100.00	25.00–100.00	12.50–93.75
> 65 years (*n* = 22)								
Median (IQR)	30.50 (9.42)	100.00 (6.25)	100.00 (91.66)	60.00 (35.00)	70.00 (42.50)	80.55 (24.99)	100.00 (0.00)	56.25 (32.81)
Mean (SD)	41.89 (26.57)	95.17 (12.78)	71.96 (43.68)	49.88 (23.91)	68.63 (23.15)	74.99 (21.73)	90.90 (24.14)	58.52 (24.81)
Range	24.00–100.00	43.75–100.00	0.00–100.00	0.00–85.00	30.00–100.00	22.22–100.00	12.50–100.00	12.50–100.00
**Years of diagnosis**								
≤ 5 years (*n* = 16)								
Median (IQR)	30.15 (13.58)	100.00 (6.25)	95.83 (97.92)	60.00 (32.50)	55.00 (30.00)	75.00 (16.67)	100.00 (21.88)	56.25 (31.25)
Mean (SD)	41.16 (24.70)	97.87 (6.45)	65.10 (45.46)	55.00 (21.25)	58.12 (22.87)	75.35 (14.34)	85.94 (27.71)	61.33 (22.96)
Range	24.00–100.00	75.00–100.00	0.00–100.00	25.00–85.00	30.00–100.00	50.00–100.00	12.50–100.00	12.50–100.00
> 5 years (*n* = 17)								
Median (IQR)	28.88 (6.9)	100.00 (3.13)	75.00 (100.00)	60.00 (41.25)	70.00 (30.00)	77.78 47.22	100.00 (0.00)	56.25 (31.25)
Mean (SD)	38.09 (23.50)	89.70 (24.60)	55.88 (46.93)	42.06 (29.41)	67.06 (24.18)	74.19 (25.98)	92.65 (19.29)	56.98 (25.95)
Range	25.00–100.00	12.50–100.00	0.00–100.00	0.00–92.50	10.00–100.00	22.22–100.00	25.00–100.00	12.50–100.00
< 10 years (*n* = 20)								
Median (IQR)	29.65 (10.22)	100.00 (6.25)	100.00 (79.17)	60.00 (41.25)	65.00 (30.00)	80.55 (26.30)	100.00 (21.87)	56.25 (29.69)
Mean (SD)	38.74 (22.52)	94.69 (13.34)	71.25 (42.45)	55.87 (25.25)	60.00 (23.62)	67.22 (17.09)	87.50 (25.33)	58.44 (23.23)
Range	24.00–100.00	43.75–100.00	0.00–100.00	0.00–92.50	30.00–100.00	44.44–100.00	12.50–100.00	12.50–100.00
≥ 10 years (*n* =13)								
Median (IQR)	29.90 (7.80)	100.00 (3.12)	8.33 (100.00)	30.00 (41.25)	70.00 (30.00)	77.78 (41.67)	100.00 (0.00)	56.25 (37.50)
Mean (SD)	40.86 (26.44)	90.86 (24.55)	43.59 (47.16)	36.73 (24.14)	66.92 (23.94)	70.94 (25.87)	92.30 (21.37)	60.10 (26.70)
Range	25.00–100.00	12.50–100.00	0.00–100.00	0.00–60.00	10.00–100.00	22.22–100.00	25.00–100.00	12.50–100.00

IQR: Inter-Quartile Range; SD: Standard Deviation; Hb1Ac: Glycated Hemoglobin; BMI: Body Mass Index; N/A: Not Applicable. * Statistically significant differences in the parameter used for comparison with the U of Mann–Whitney.
